# Estimation of the Burden of Ischemic Heart Disease in the Tabasco Population, Mexico, 2013–2021

**DOI:** 10.3390/ijerph22030423

**Published:** 2025-03-13

**Authors:** Jesús Josué Cárdenas-Anguiano, Sergio Quiroz-Gomez, Crystell Gudalupe Guzmán-Priego, Karla del Socorro Celorio-Méndez, Manuel Alfonso Baños-González, Alejandro Jiménez-Sastré, Guadalupe del Carmen Baeza-Flores, Jorda Aleiria Albarran-Melzer

**Affiliations:** 1 Health Sciences Academic Division (DACS), Juarez Autonomous University of Tabasco (UJAT), Villahermosa 86040, Mexico; 172e45114@egresados.ujat.mx (J.J.C.-A.); karla.celorio@ujat.mx (K.d.S.C.-M.); mbg04677@docente.ujat.mx (M.A.B.-G.); alejandro.jimenez@ujat.mx (A.J.-S.); jorda.albarran@ujat.mx (J.A.A.-M.); 2 Cardiometabolism Laboratory, Research Center, Health Sciences Academic Division (DACS), Juarez Autonomous University of Tabasco (UJAT), Villahermosa 86040, Mexico; crystell.guzman@ujat.mx (C.G.G.-P.); guadalupe.baeza@ujat.mx (G.d.C.B.-F.)

**Keywords:** disability-adjusted life years, burden of disease, ischemic heart disease

## Abstract

Introduction: The burden of disease measures the total impact of diseases on a population, considering incidence, prevalence, disability, and premature mortality. This study analyzes the burden of ischemic heart disease (IHD) in Tabasco, Mexico, from 2013 to 2021. Ischemic heart disease has a significant incidence of 21,203,479 cases worldwide, and nationally (inside Mexico) a total of 221,747 cases, with more than 9,137,791 deaths due to this pathology globally. Objective: To analyze the burden of ischemic heart disease in Tabasco, Mexico, during the 2013–2021 period. Methods: An observational, descriptive, longitudinal, and retrospective study was conducted in Tabasco. The study population consisted of 2,402,598 people according to INEGI, with a sample of 927,000 adults (462,000 men and 465,000 women). Data were used from the General Directorate of Health Information, IHME, and the World Bank. Analyses were performed in Microsoft Excel, calculating measures of central tendency, dispersion, and Disability-Adjusted Life Years (DALYs). Results: The DALYs in the adult population of Tabasco were: 2013—23,932; 2014—28,132; 2015—30,197; 2016—30,683; 2017—31,839; 2018—38,599; 2019—40,046; 2020—42,307; and 2021—55,723, totaling 297,576 DALYs from 2013 to 2021. Discussion: Ischemic heart disease increased in incidence and mortality in both men and women during the years analyzed. The increase in DALYs indicates a greater impact of ischemic heart disease in Tabasco compared to countries like Costa Rica. Conclusion: The burden of ischemic heart disease from 2013 to 2021 represents a significant loss of quality and years of life in the population of Tabasco, Mexico.

## 1. Introduction

The burden of disease is defined as a comprehensive and quantitative measure of the total impact of diseases on a population. This evaluation encompasses not only the incidence and prevalence of diseases but also the associated disability, premature mortality, and other factors affecting quality of life. It is used to understand the relative burden of different diseases and health conditions in a community, providing valuable information for planning and resource allocation in public health. The burden of disease is often expressed in terms of Disability-Adjusted Life Years (DALYs), which allows for the comparison of the impact of various diseases by considering both mortality and years lived with disability [1].

In this context, the burden of disease associated with ischemic heart disease (IHD) emerges as a global concern of considerable magnitude in public health. It transcends physical implications, extending to emotional and economic spheres. Individuals experiencing IHD often face long-term consequences such as decreased quality of life and the potential for disabilities. In Mexico, IHD has become one of the leading causes of mortality. In 2013, this disease claimed the lives of 88,775 people nationwide, with 1451 deaths occurring in the state of Tabasco. The incidence of cases that year was alarming, with 304,286 new diagnoses across the country and 5460 in Tabasco. The national prevalence reached 2,578,398 cases, while 44,267 people were affected in Tabasco. The situation worsened significantly in 2021, with the number of deaths in Mexico rising to 129,103, of which 2441 occurred in Tabasco. The national incidence skyrocketed to 412,013 cases, with 7293 new diagnoses in Tabasco. The prevalence also showed a worrying increase, with 3,513,288 cases nationwide and 59,995 in Tabasco [2].

### 1.1. Ischemic Heart Disease

Annually, over 1.7 million lives are lost to cardiac ischemia in Europe, accounting for a troubling 20% of all deaths. In the United States, it is the cause of approximately 30% of deaths among those over 35 years old [3]. Affecting 126 million people worldwide, or about 1.72% of the global population, this disease continues to spread. Projections suggest that the current prevalence of 1655 cases per 100,000 could rise to over 1845 by 2030, with Eastern Europe experiencing the highest rates [4].

The economic impact is equally concerning. In 2010, the global cost of cardiovascular diseases was approximately 863 billion dollars, and it is expected to exceed one trillion dollars by 2030. In low- and middle-income countries, the total cost of care for ischemic heart disease accounts for 10% of total health expenditure, highlighting the need for more efficient and sustainable management strategies [5].

The burden of morbidity associated with ischemic heart disease is also profound. Cardiovascular diseases are responsible for about one-third of all deaths globally [6]. An increasing number of survivors live with chronic disabilities and significantly impaired quality of life, which emphasizes the necessity for effective therapeutic approaches and preventive measures [7].

Within the spectrum of ischemic heart disease are acute coronary syndromes, which include three main entities:

1. Unstable Angina: Characterized by chest pain of recent onset, increasing in frequency, duration, or intensity in a patient with previous stable angina, or pain occurring at rest. In an electrocardiogram (ECG), unstable angina may show ST-segment depression or T-wave inversion, but without ST-segment elevation. There is no elevation of myocardial necrosis biomarkers [8].
2. Non-ST-segment Elevation Myocardial Infarction (NSTEMI): Manifested by chest pain like that of unstable angina, but accompanied by elevated myocardial necrosis biomarkers such as troponin T and troponin I. An ECG may show ST-segment depression or T-wave inversion, but without ST-segment elevation [9].
3. ST-segment Elevation Myocardial Infarction (STEMI): Presented with persistent and severe chest pain, and an ECG shows ST-segment elevation in at least two contiguous leads, with specific criteria depending on the patient’s age and sex. This type of infarction requires rapid intervention to restore coronary blood flow [8].

For the treatment of ischemic heart disease, there are two main options: catheterization and fibrinolysis. The choice between the two mainly depends on the available time for timely intervention [10]. If more than two hours are available at a center with the capacity to perform catheterization, this procedure is preferable. It involves placing a stent through an artery, usually the femoral artery, to restore blood flow to the heart by removing the obstructing clot. On the other hand, if less time is available, fibrinolysis, a pharmacological treatment that aims to dissolve the clot, is chosen [11].

### 1.2. Essential Hypertension

Arterial hypertension is a chronic-degenerative disease with high prevalence and incidence worldwide, known as the “silent killer” due to its lack of symptoms in the early years of evolution. It is characterized by a sustained elevation of blood pressure against the arterial walls, according to the guidelines established by the American Heart Association (AHA). It affects approximately one-third to one-half of adults in the United States [12], where most adult hypertension cases, around 90%, are primary hypertension, also called essential hypertension. Additionally, an increase in its prevalence among children and adolescents has been observed [13].

Along with conditions such as diabetes mellitus and hypercholesterolemia, it promotes the formation of atherosclerotic plaques. Additionally, hyaline deposition is the mechanism by which the disease progresses, narrowing the arterial lumen and increasing blood pressure. Over time, this mechanism produces complications, such as strokes, acute myocardial infarctions, chronic kidney disease, and hypertensive retinopathy [14].

Hypertension is classified into several grades according to the AHA: Grade 1 is defined by systolic pressure in the range of 130–139 mm Hg and diastolic pressure of 80–89 mm Hg. On the other hand, Grade 2 hypertension is characterized by readings equal to or greater than 140 mm Hg for systolic pressure and 90 mm Hg for diastolic pressure. Most of the time, hypertension tends to be asymptomatic until complications arise from organ damage or there is an acute increase in blood pressure [15,16].

Treatment, according to European guidelines, generally begins with dual therapy. In the case of the AHA, it depends on the blood pressure levels. With Grade 1, treatment with monotherapy is established, and with Grade 2, dual therapy is initiated [17]. The combination of ACE inhibitors and ARBs is contraindicated [18] and other treatment options may be considered if necessary. First-line medications include ACE inhibitors, ARBs, hydrochlorothiazide, and calcium channel blockers [19].

### 1.3. Type 2 Diabetes Mellitus

Type 2 diabetes mellitus (T2DM) is a chronic and degenerative disease that progresses over time and has no cure. The prevalence of diabetes in 2016 was 9.4% (CI95% 8.3–10.8%), which translated to just over 6.4 million people diagnosed with diabetes in Mexico [20]. In 2018, approximately 13.3 million adults had diabetes, representing 16.8% of the population. By 2020, this figure slightly decreased to 12.8 million, equivalent to 15.7%. It is important to note that in 2018, 38% of total diabetes cases were undiagnosed, and 165,000 patients belonged to the age group between 20 and 39 years [21]. Ignacio Conget, in his article published by the University of Barcelona, titled “Diagnosis, Classification, and Pathogenesis of Diabetes Mellitus”, defines it as a metabolic abnormality characterized by the persistence of elevated blood glucose levels, accompanied to varying degrees by alterations in the metabolism of carbohydrates, proteins, and fats [22]. Over time, the excess unabsorbed glucose can cause damage to various organs in the human body, such as the kidneys, brain, heart, and retina. Although it may initially be asymptomatic, type 2 diabetes can lead to severe complications, such as chronic kidney disease, diabetic retinopathy, chronic arterial insufficiency, diabetic foot, and cerebrovascular events [23].

The approach to treating type 2 diabetes mellitus varies according to the level of glycated hemoglobin diagnosed in the patient. If glycated hemoglobin is between 6.5 and 10, according to the latest ADA guidelines in 2024, two medications stand out as first-line options: GLP-1 analogs and SGLT-2 inhibitors. Examples are liraglutide and empagliflozin, respectively. GLP-1s are cardioprotective and help reduce weight in patients with diabetes and obesity or overweight, making them a valuable option not only for controlling blood sugar but also for addressing weight loss, while empagliflozin protects the kidneys, reducing the risk of chronic kidney disease. Therefore, they have established themselves as the main treatments for diabetic patients [24,25]. However, in Mexico, for economic reasons, most diabetic patients do not have access to these treatments. Metformin, available in 850 mg and 500 mg presentations, is the basis of treatment for most Mexicans due to its low cost of just 3600 Mexican pesos annually [26].

The association between type 2 diabetes and ischemic heart disease is largely related to protein glycation. This occurs when sugars bind to proteins, which can cause deposits in blood vessels, reducing their diameter and increasing systemic resistance, which, in turn, can trigger heart failure and, eventually, ischemic heart disease. Therefore, it is essential to implement effective preventive measures to reduce the incidence of diabetes mellitus [22].

### 1.4. Obesity

Obesity is recognized as a chronic degenerative disease. Previously defined only as a physical state, it is now considered a disease characterized by a body mass index (BMI) over 30 or an abdominal circumference greater than 102 cm in men and 88 cm in women. This is because abdominal fat accumulation has been identified as an additional risk factor for the development of comorbidities [27]. This paradigm shift recognizes obesity as an independent disease. However, continuous research and training in the field of obesity must be encouraged, focusing on developing more effective and personalized interventions. Despite fundamental advances in the battle against obesity, a highly effective treatment has not yet been found [28].

In Mexico, 36.9% of adults suffer from obesity, while 38.3% are overweight. The prevalence of abdominal obesity in people over 20 years old is 81.0% [29]. Although the prevalence of abdominal obesity is high, especially in countries like the United States and Mexico, the lack of depth in its study makes it difficult to fully understand its origin. Factors such as a high-calorie diet, lack of physical activity, and mental health problems such as depression and anxiety are related to its occurrence. Additionally, the lack of nutritional education provided to the population plays a fundamental role in empowering companies to manipulate people’s eating habits, lifestyle, and the development of this disease [30] While there are other possible causes, such as hypothyroidism and certain medications, obesity as a direct consequence of these is less common.

Previously, obesity was perceived as a challenge of self-control, but it is now understood that there are dysfunctions in the central nervous system pathways that regulate satiety and appetite. This understanding has led to the realization that a comprehensive approach, including lifestyle modifications and individualized medical and pharmacological interventions, is more effective than each component [31,32,33,34,35].

Pharmacological treatment plays an important role in managing obesity, especially in cases where dietary and lifestyle interventions alone have not been effective. Although pharmacotherapy presents promising options, it faces challenges in terms of long-term efficacy and potential side effects [36]. However, advances in understanding the neurobiology of obesity are revealing new pharmacological strategies that could improve treatment outcomes [37]. These advances have led to the FDA approval of several medications for obesity treatment [38]. Approved anti-obesity medications include orlistat, SGLT2 inhibitors, GLP-1 analogs, and drug combinations such as phentermine/topiramate. Orlistat, for example, works by reducing fat absorption in the intestine, while GLP-1 analogs, such as semaglutide, work by decreasing appetite and promoting satiety. Other approved medications also follow different mechanisms, but these mechanisms are not yet well understood [39].

Despite their benefits, pharmacological treatment of obesity presents various challenges, including adherence to the regimen, potential side effects, and economic costs. In this last challenge, phentermine stands out as an economical option, but GLP-1 analogs, such as liraglutide, are considered more effective and offer additional cardiac protection, although their high-cost limits access for most of the Mexican population.

The Organization for Economic Cooperation and Development ranked Mexico with the highest mortality due to ischemic heart disease [40]. First, ischemic heart disease is the leading cause of death, responsible for 16% of all deaths worldwide. Since 2000, the greatest increase in deaths corresponds to this disease, which has increased from more than 2 million deaths in 2000 to 8.9 million in 2019, which makes it a priority problem for health systems. This is reflected in the increase in DALYs from the year 2000, with 1661 per 100,000 inhabitants, to 1795 per 100,000 inhabitants in 2019. Mexico went from belonging to the first quintile to the fourth quintile [41], which makes evident the need to study the historical evolution and the current situation of our population in this regard.

The COVID-19 pandemic has had a profound effect on global cardiovascular health. Studies have shown that SARS-CoV-2 infection can cause direct damage to the cardiovascular system, increasing the risk of complications such as myocarditis, arrhythmias, and thrombotic events [42]. In addition, an increased incidence of myocardial infarction and stroke has been observed in patients with COVID-19, even in those without prior risk factors [43].

The indirect impact of the pandemic on cardiovascular health has also been substantial. Containment measures and fear of contagion led to a decrease in seeking medical care for acute and chronic cardiovascular conditions, resulting in delayed diagnoses and worse outcomes. In terms of life expectancy, COVID-19 has had an unprecedented impact. A 1.87-year decline in life expectancy has been reported in the United States between 2018 and 2020, the largest drop since World War II. This decline was even more pronounced in minority and low-income populations, exacerbating existing health disparities [44].

Globally, a study by Aburto et al. [41] found that life expectancy declined in 31 of 37 countries analyzed in 2020, with reductions of more than one year in 22 countries. These losses in life expectancy are unprecedented in peacetime in the modern era and reflect both direct deaths from COVID-19 and excess mortality associated with the disruption of health systems and social determinants of health [45]. 

This research aims to analyze the burden of ischemic heart disease in the state of Tabasco, Mexico, from 2013 to 2021. Through rigorous interpretation of the available data, the study seeks to understand the context and establish a foundation for future projects aimed at improving the public health landscape in the country and particularly in the state of Tabasco.

## 2. Materials and Methods

### 2.1. Study Design

This study is observational, descriptive, longitudinal, and retrospective, conducted in the state of Tabasco, Mexico, during the period from 2013 to 2021. Available data were analyzed to assess the burden of ischemic heart disease.

### 2.2. Study Population

The study universe encompassed 2,402,598 people according to the National Institute of Statistics and Geography [42]. A representative sample of 927,000 adults (over 18 years old) was taken, of which 462,000 were men and 465,000 women.

### 2.3. Data Collection and Preparation for Analysis

Data were obtained from the General Directorate of Health Information (DGIS) for the years 2013–2021 (http://www.dgis.salud.gob.mx/contenidos/basesdedatos/da_defunciones_gobmx.html, accessed on 1 July 2024), as well as from the Institute for Health Metrics and Evaluation (IHME) (https://www.healthdata.org/data-tools-practices/interactive-data-visuals, accessed on 1 July 2024) and the World Bank (https://datos.bancomundial.org/indicator/SP.DYN.LE00.IN?locations=MX, accessed on 1 July 2024). These data are open access to anyone who wishes to consult them and include sufficient information to obtain incidence, prevalence, mortality, and life expectancy [46,47,48].

Measures of central tendency and dispersion were performed, as well as the calculation of disability-adjusted life years (DALYs). Frequency tables were generated to present the data in a structured manner, and bar and line graphs were created to visualize the results. Data checks and formulas were performed to ensure accuracy, and the results were interpreted in the context of the theoretical framework and the objectives of the study.

To calculate the DALYs, the years lost due to premature death were first determined by multiplying the number of cases of death by life expectancy. Subsequently, years of life lived with disability were calculated using the duration of the disease, defined as life expectancy minus the average age of disease onset [49]. This value was subtracted from life expectancy and multiplied by the number of disabilities, also known as disease burden, obtained from the Daly Calculator [48]. This was used to obtain the years of life with disability per disease, which were multiplied by the incidence of the disease, obtained from the Institute for Health Metrics and Evaluation [50], to obtain the years of life with disability per disease. Finally, years of life lost due to premature death and years of life lived with disability were summed to obtain DALYs. In addition, a secondary calculation of DALYs was performed using data from the Global Burden of Disease Study, keeping the same parameters and calculations, but obtaining the cases of deaths directly from the platform of the aforementioned study.

Survival was calculated by subtracting the number of cases of deaths from the number of new cases of the disease (incidence). The survival percentage was calculated by group and age, using data from the years 2013 and 2021, the first and last year of the analysis period. A secondary analysis was also performed using the cases of deaths reported by the Global Burden of Disease Study.

### 2.4. Calculation of Disability-Adjusted Life Years (DALYs)

The calculation of Disability Adjusted Life Years (DALYs) was performed in accordance with a specific methodology. Initially, Years of Life Lost (YLL) were determined, which were obtained by multiplying the number of deaths by the life expectancy at the time of disease onset. Subsequently, Years Lived with Disability (YLD) were calculated by subtracting the average age at disease onset from life expectancy and multiplying this value by the weight of the disease, obtained through the DALY Calculator. The total burden of disease was then estimated by adding the YLL and YLD values, which allowed the calculation of the total DALYs.

### 2.5. Data Analysis

Measures of central tendency were used to analyze the data. Additionally, frequency tables and graphs were generated to visualize the results. Calculations were performed using Microsoft Excel, and formulas and data were verified to ensure accuracy (Table S1).

The sensitivity analysis aims to evaluate the robustness of the results obtained in the calculation of Years of Life Lost (YLL), an indispensable variable for the calculation of DALYs, due to ischemic heart disease in Tabasco, Mexico. For this purpose, we have modified the two main variables involved in the calculation of DALYs: number of deaths and life expectancy. These variables were selected because of their direct influence on the DALY formula, and to ensure that the model results are consistent in the face of possible fluctuations in the data.

Percentage variations were applied to the number of deaths and life expectancy variables in ranges of ±5%, +10%, and +5%, +5%. These variations were chosen to represent both incremental and decremental changes in mortality rates and life projections, allowing us to observe how these changes affect the DALY outcomes. The analysis was performed separately for men and women, and the results obtained were compared with the baseline scenario, without modifications.

### 2.6. Ethical Considerations

The study was conducted in accordance with the principles of the Declaration of Helsinki. The research was approved by the Research Ethics Committee of the Juárez Autonomous University of Tabasco, with code JI-LCT, complying with current regulations established in NOM-012-SSA3-2012. All necessary measures were taken to protect the privacy and confidentiality of participants’ data, ensuring that information was handled ethically and respectfully.

### 2.7. Inclusion and Exclusion Criteria

Inclusion Criteria:

- Unstable angina (DGIS I200)
- Unspecified angina pectoris (DGIS I209)
- Acute transmural myocardial infarction of the anterior wall (DGIS I210)
- Acute transmural myocardial infarction of the inferior wall (DGIS I211)
- Acute transmural myocardial infarction of other sites (DGIS I212)
- Acute transmural myocardial infarction of unspecified site (DGIS I213)
- Acute subendocardial myocardial infarction (DGIS I214)
- Acute myocardial infarction not otherwise specified (DGIS I219)
- Subsequent myocardial infarction of unspecified part (DGIS I229)

Exclusion Criteria:

- Minors
- Records with incomplete or missing information
- Cases reported outside the 2013–2021 period
- Other forms of unspecified acute or chronic ischemic heart disease (DGIS I248, I249, I259, I258, I256, I255).

## 3. Results

The study population was characterized as follows: 927,000 inhabitants of Tabasco were selected, with 462,000 men and 465,000 women, over 18 years. This distribution shows a slight majority of women (51.1%) over men (48.9%). The average age of Tabasco’s adult population is approximately 41.9 years, with men being an average of 39.7 years old and women an average of 44.1 years old.

### 3.1. Incidence and Mortality Trends

**Figure 1:**
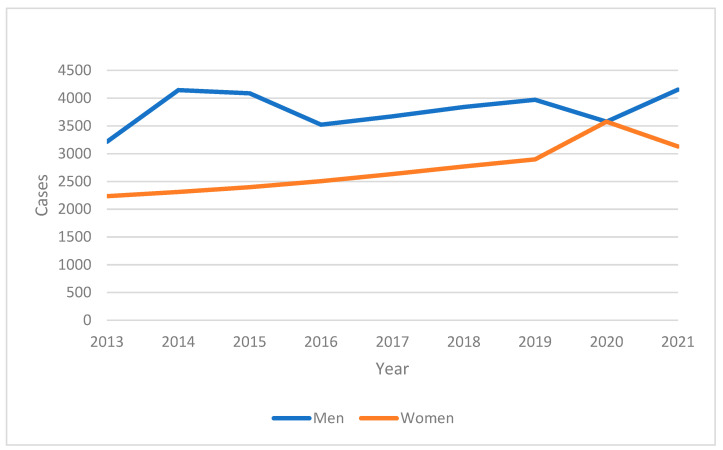
Incidence in adult population by year and sex in the period 2013–2021 in Tabasco.

From 2013 to 2021, the incidence of ischemic heart disease (IHD) in the adult population of Tabasco showed a concerning upward trend. The annual incidence rates, as shown in Figure 1, indicate that the number of new cases increased consistently over the study period. This increase was more pronounced in males compared to females, suggesting possible gender-specific risk factors or health behaviors influencing the disease’s prevalence.

**Figure 2:**
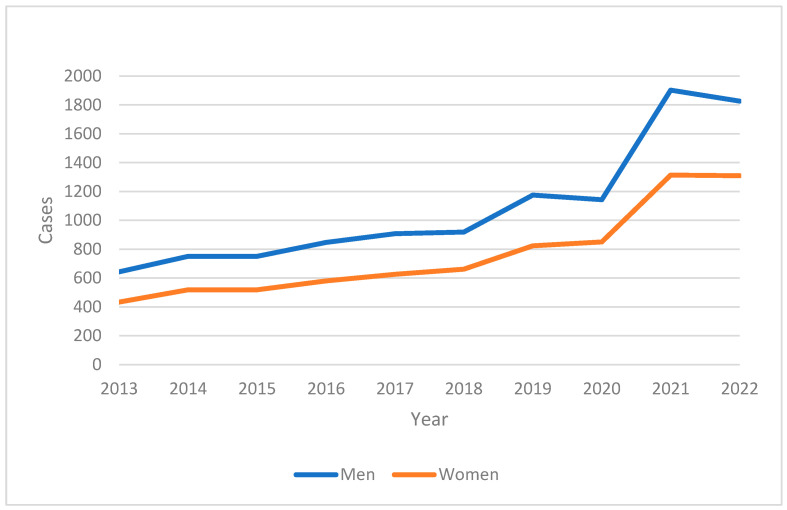
Number of deaths in adult population in the period 2013–2021 in Tabasco.

The mortality data, illustrated in Figure 2, reveal a significant rise in the number of deaths attributed to IHD during the same period. Notably, there was a sharp increase in 2020, which may be associated with the impact of the COVID-19 pandemic on healthcare systems and patients’ health-seeking behavior.

### 3.2. Survival Analysis

**Figure 3:**
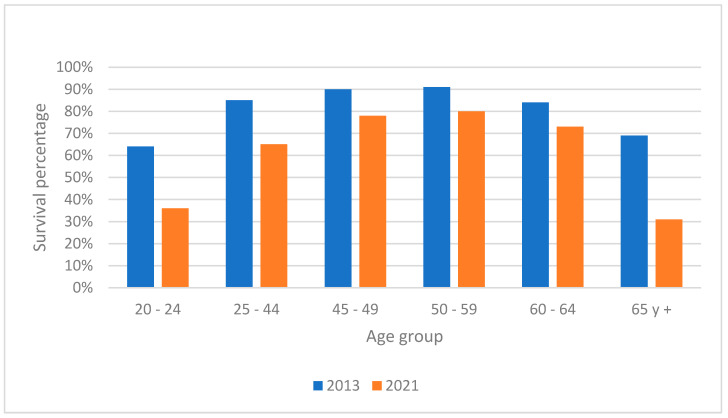
Survival comparison of 2013 and 2021 for men by age group in Tabasco.

**Figure 4:**
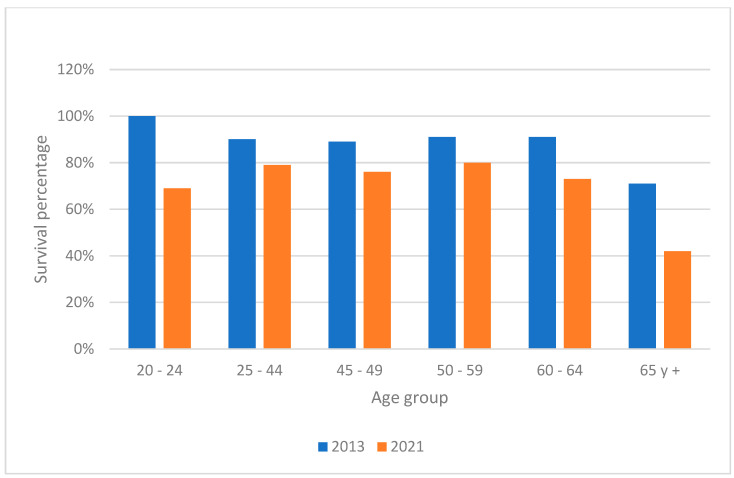
Survival comparison of 2013 and 2021 for women by age group.

Survival rates were calculated by subtracting the number of deaths from the number of new cases, with results stratified by age and gender for the years 2013 and 2021 (Figure 3 and Figure 4). The data showed a noticeable decline in survival rates over the years, particularly in younger age groups, suggesting a deterioration in either the efficacy of treatments or the overall health status of the population. This trend was more severe in males compared to females.

### 3.3. Disability-Adjusted Life Years (DALYs)

**Figure 5:**
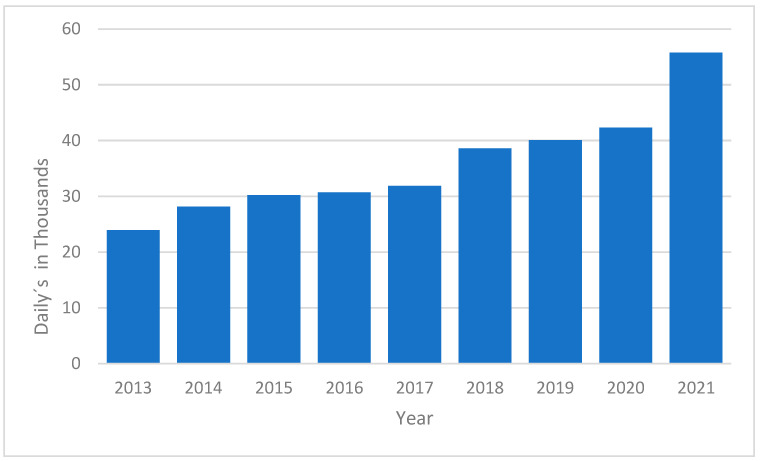
DALYs in the adult population per year in the 2013–2021 period. The results of the sensitivity analysis indicate that variations in the input variables do not have a significant impact on the DALY values. Specifically, changes in the number of deaths, even with a variation of +10%, generated minor fluctuations in total DALYs. Similarly, changes in life expectancy produced marginal changes, with lower variation in all scenarios tested.

The burden of IHD was further quantified using Disability-Adjusted Life Years (DALYs), which combine years of life lost due to premature mortality and years lived with disability. The calculation of DALYs involved determining the years lost to premature death and the years lived with disability, adjusted by the disability weight specific to IHD. The total DALYs increased significantly from 2018 onward, with a marked peak in 2020 and 2021. This increase aligns with the rise in mortality during the COVID-19 pandemic, underscoring the heightened impact of the disease during this period (Figure 5).

The results suggest that the model is robust to fluctuations in the death and life expectancy data, which reinforces the validity of the results presented. The fact that percentage variations in the key variables do not significantly impact the DALY values indicates that the conclusions of the study are stable and are not influenced by minor errors or uncertainties in the data. This stability is particularly important in the context of public health, where decisions must be based on reliable estimates that are robust to moderate changes in input conditions.

## 4. Discussion

The incidence and mortality of ischemic heart disease (IHD) in both men and women shows an upward trend, especially in the later years of the analyzed period. This increase may be related to risk factors such as an aging population, lifestyle changes, and possible deficiencies in the prevention and management of the disease, the number of deaths due to IHD rose sharply, particularly in 2020, likely exacerbated by the COVID-19 pandemic. The incidence in Tabasco (304.1 in 2021) is significantly higher than in Costa Rica (84 in 2017), suggesting a higher prevalence of risk factors in Tabasco [51]. Similarly, the incidence in Tabasco is higher compared to Colombia in 2021 (90.2) [52], indicating a greater burden of disease in this region during recent years.

The mortality rate in Tabasco (130.9 in 2021) is considerably higher than in Costa Rica and Ecuador (68.5 in 2019–2021) [51,53], which may reflect differences in access to and in the quality of healthcare [46]. The notable increase in mortality in Tabasco during 2020 (135.9) could be associated with the impact of the COVID-19 pandemic. According to Mukkawar et al., the pro-inflammatory cytokine cascade triggered by this disease could exacerbate cardiovascular diseases [54].

The reduction in average survival rates in both men and women from 2013 to 2021 suggests a deterioration in treatment outcomes or an increase in the severity of cases. The more pronounced reduction in younger age groups might indicate growing vulnerability in these populations. According to data from the Institute for Health Metrics and Evaluation (IHME), overall survival decreased; however, compared to the present research, it was higher in both men and women (60% vs. 72% in men; 69% vs. 80% in women). The elderly (over 65 years) and the 50–59 age group were the least likely to survive throughout the study period, with the lowest survival rate in 2021 among men aged 20–24.

The present study explores the impact of the pandemic on global health, considering both the direct mortality resulting from the virus and its indirect effects on health systems and the quality of life of populations. The findings of the present study demonstrate a substantial increase in DALYs due to the pandemic, thus classifying it as one of the primary causes of DALYs in 2020. This finding aligns with global trends, as evidenced by studies showing particular impacts in countries such as Mexico and Portugal [55].

A recurring theme in all studies is the disproportionate impact on vulnerable populations and the exacerbation of existing health inequalities. This is observed both at the national level in Mexico and in the comparison between countries in global and regional studies [56].

In this sense, the indirect effect of the pandemic on the increase in DALYs due to the interruption of health services for other conditions has been presented. This suggests that the total impact of the pandemic on DALYs may be even greater than can be directly attributed to COVID-19 [57]. Scientific evidence has shown that socioeconomic factors such as income, education, and access to health care are directly associated with the incidence of and mortality from ischemic heart disease. Populations with lower levels of education have a higher prevalence of modifiable risk factors, which increases the burden of disease. In addition, inequalities in access to health services affect the control of hypertension and dyslipidemia, the main causes of the disease. Although this study focuses on estimating the burden of disease, the inclusion of these socioeconomic determinants in future research will provide a better understanding of their impact and will strengthen the formulation of health policies [58].

The study emphasizes the need for comprehensive interventions that address both risk factors and improvements in healthcare services and medical staff training. Promoting cardiovascular health and preventing cardiovascular diseases through adequate public health policies and programs are essential to reduce the burden of IHD in Tabasco and improve the quality of life for affected individuals. It is important to note that gaps or inconsistencies may exist in the historical data used in the study that limit the analysis of the information obtained; the accuracy and completeness of the historical records may vary significantly. These limitations do not invalidate retrospective studies, but were carefully considered in the design, analysis, and interpretation of the presented results.

## 5. Conclusions

The results show an increase in the incidence and mortality of the disease, with a notable rise in recent years due to possible factors such as population aging, changes in lifestyles, and deficiencies in prevention and treatment.

The significant increase in Disability-Adjusted Life Years (DALYs) from 2018 onward reflects a greater impact of IHD on the disease burden. The high DALY burden in Tabasco (297, 576 years between 2013 and 2021) indicates a substantial loss of quality of life and years of life due to IHD in this region. In terms of survival, a notable decline was observed in both sexes from 2014 to 2021, with more pronounced reductions in younger age groups, which could indicate increasing vulnerability and deterioration in treatment efficacy. This trend is most acute in younger men and older women, highlighting an urgent need for improved disease management and prevention strategies. The significant increase in Disability Adjusted Healthy Life Years (DALYs) reflects an increased burden of disease in the population, with marked increases starting in 2018 and an acute impact in 2020 and 2021, coinciding with the pandemic.

Our sensitivity analysis demonstrated that the model used to calculate the DALYs due to ischemic heart disease is robust to variations in the number of deaths and life expectancy. These findings support the reliability of the results presented in the study, providing further confidence in the robustness of the projections and the usefulness of DALYs as a tool for health policy planning.

The results of this investigation point to an alarming trend of increasing incidence and mortality from ischemic heart disease, accompanied by a decrease in survival and an increase in the burden of disease as measured by DALYs. These findings emphasize the urgency of implementing more robust public health policies focused on the prevention, management, and treatment of ischemic heart disease, especially in vulnerable populations and during health emergencies. They also highlight the need for gender- and age-differentiated approaches to effectively address the observed health disparities.

### Recommendations

Given the increase in the burden of disease and its impact on morbidity and mortality, it is imperative to strengthen primary and secondary prevention strategies. Based on the analysis conducted, the following specific interventions are proposed, aligned with international recommendations and adapted to the national context:

Implementation of a comprehensive model for the screening and control of cardiovascular risk factors at the first level of care.

- Incorporate risk stratification algorithms based on validated scales (SCORE2, Framingham) for the early identification of individuals at high risk.
- Expand coverage of ambulatory blood pressure and lipid profile monitoring in high-risk populations, ensuring their inclusion in national public health programs.
- Optimize therapeutic adherence through telemedicine systems and remote follow-up, integrating automated reminders for taking antihypertensive drugs and statins.

Strengthening regulation and fiscal policies for the reduction of modifiable risk factors.

- Increase the tax burden on ultra-processed products that are high in sugars and saturated fats, with a budget allocation aimed at cardiovascular prevention programs.
- Regulate the advertising of calorie-dense foods to minors, following successful intervention models implemented in countries such as Chile and the United Kingdom.
- Expand front-end warning labeling, integrating cardiovascular risk indicators in products that are widely consumed in the Mexican diet.

Strengthen the Primary Health Care Model with a focus on cardiovascular diseases.

- Train health personnel in the detection and management of risk factors through updated, evidence-based clinical practice guidelines (ESC, ACC/AHA).
- Implement an efficient referral and counter-referral system that guarantees continuity of care between first level units and high specialty hospitals.
- Promote the integration of community interventions through health promoters trained in cardiovascular prevention strategies.

Monitor and evaluate the impact of interventions through robust epidemiological analyses.

- Incorporate the periodic calculation of DALYs and other disease burden indicators into national epidemiological surveillance systems, allowing the evaluation of trends and increasing the effectiveness of implemented interventions.
- Establish an observatory of chronic noncommunicable diseases with access to open databases for researchers and decision makers.
- Promote collaboration with academic institutions and international organizations for the implementation of population-based studies that analyze the effectiveness of preventive policies in different population subgroups.

These initiatives aim to improve the prevention, management, and treatment of ischemic heart disease, addressing observed health disparities and promoting a culture of preventive health from childhood.

## 6. Study Limitations

The availability of new data on incidence and mortality limits the ability to conduct a more updated analysis.

## Data Availability

Mortality data used in this study were obtained from the General Directorate of Health Information (DGIS) and are available at the following link: https://www.gob.mx/salud/acciones-y-programas/direccion-general-de-informacion-en-salud-dgis (accessed on 12 February 2024). Life expectancy data were obtained from the World Bank and are available at the following link: https://data.worldbank.org/indicator/SP.DYN.LE00.IN?locations=MX (accessed on 12 February 2024). Incidence data used in this study were obtained from the Insitute for Health Metrics and Evaluation (IHME) and are available at the following link: https://vizhub.healthdata.org/gbd-results/ (accessed on 1 July 2024).

## Supplementary Materials

The following supporting information can be downloaded at: https://www.mdpi.com/article/10.3390/ijerph22030423/s1, Table S1: Sensitivity analysis table.
